# Supplementary material for: Understanding the implication of direct health facility financing on health commodities availability in Tanzania
Source: PLOS Glob Public Health. 2023 May 8;3(5):e0001867. doi: 10.1371/journal.pgph.0001867 (PMC10166559; doi:10.1371/journal.pgph.0001867)
Supplement: Attachment — Submitted filename: Reviewer comments_All.docx [file pgph.0001867.s001.docx]

| **REVIEWER NO 1** | | | |
| --- | --- | --- | --- |
| **SN.** | **Reviewers Comments** | **Response** | **Page** |
|  | **Several comments** | | |
| 1. | It would be helpful to explain how finance resources were allocated prior to DHFF in the introduction of the paper, so the policy implications of DHFF will be better understood for readers without background knowledge. | Thanks, a statement…. “Prior to the introduction of DHFF, all planning and budgeting was being done at the council level, including procurement and distribution of health commodities. DHFF introduction has enabled health facilities to plan and budget according to their demands. The health facilities have autonomy in procurement of health commodities”… has been added | **Pg. 3** |
| 2. | How was the key variable in Figure 1, Average Health Commodity Availability defined, is this index an average of the availability of all 312 health commodities? | Thanks, Figure one is the average availability of 312 health commodities. The term of the 312-tracer medicine has been added to increase clarity…. | **Pg.9** |
| 3. | In Figure 1, there was an increase in Average Health Commodity Availability from 2015/16 to 2016/17. A discussion on the pre-DHFF trend of health commodity availability would be helpful for the reader to decompose the policy effect of DHFF from the baseline increasing tread. | Thanks, this paper is set as a baseline upon which future studies could decompose the availability of health commodities in relation to funding. A statement has been added in the discussion to highlight the limitation …… “There was no financial availability by facility levels rom prior to DHFF in 2015/16 to 2016/17, this limits the discussion of the effect of the health financing prior to DHFF policy. | **Pg.13** |
| 4. | I recommend changing the type of the Figure 2 chart to a clustered column chart. The current format of the chart is misleading, the numbers does not sum up to 100% in each column. | Thanks, Figure 2 has been changed to a cluster column | **Pg.10** |
|  | **Minor comments** |  |  |
| 1 | The abbreviation "MSD" in the last paragraph on Page 5 is not included in the List of Abbreviations or explained in the article. | Thanks, the long form of MSD i.e., Medical Stores Department (MSD) has been added | **Pg.5** |
| 2 | What is the currency unit in Table 1? | The currency is in USD$ |  |
| 3 | In addition, I would recommend checking the grammar. For example, on Page 11, the second paragraph, a period is needed after the first sentence. In the same paragraph, the phrase "The low adherence to health commodity cost sharing guidelines" is not a complete sentence. | Thanks, a thorough copy editing has been done, the sentence on Pg 11 now reads… ***the guideline recommendation of spending a minimum of 50% to procure health commodities.*** | **Pg. 9** |

**REVIEWER 2**

| **SN.** | **Reviewers Comments** | **Response** | **Page** |
| --- | --- | --- | --- |
|  | **Minor Comments** | | |
| 1 | The main results of the study was derived using Excel to summarize the health commodity budgeting, expenditure and availability on yearly base. No statistical inference can be derived. In another way, readers cannot draw convincing conclusion from the results. | Thanks, we have added stastical analysis on the correlation of health commodity expenditure and the availability of medicine by facility levels….. also added the stastical analysis on the availability of health commodity by geographical allocation of the health facility | **Pg. 11** |
| 2 | There is not enough background information, theoretical setup and discussion for the readers to understand how the DHFF solve the existing problem and how it can facilitate the road towards UHC in Tanzania. | Thanks, the linkage of DHFF and UHC has been underpinned in the background…. “Health financing arrangements geared towards ensuring availability of adequate financial resources and efficiency in resource utilization is crucial in achieving intended goals of UHC. According to the World Health Organization (WHO) the inefficient use of resources hinders rapid movement towards UHC. Efficient resource use reduces waste, improves the ability of health systems to provide quality services and improves population health. The availability of essential health commodities medicines is a core for any health system working to achieve UHC. Strong health systems are advocated as a tool for accelerating UHC The current improvement in the financial management information systems in Tanzania is key in the allocation and tracking of financial resources for essential health commodities towards UHC. Equitable access to essential health commodities is an important component for a well-functioning health system. Monitoring and evaluation of reforms that are pitched on improving availability of health commodities is of paramount importance towards realization of the UHC policy. However, to date, there is paucity of documented literature in Tanzania with regards to the effectiveness of DHFF reforms in improving the availability of health commodities in primary health care facilities | **Pg 4** |
|  | **Major Comments** | | |
|  | **Background** | | |
| 1 | The authors should provide more information on the current healthcare system in Tanzania and the existing challenges/issues in the healthcare system. In particular, the issues related to health care financing should be explained. This can help readers better understand the rationale of introducing DHFF. | Additional information has been added in the background to improve readability and understanding of the health sector financial management systems in Tanzania…. “Health services delivery services in Tanzania is overseen by two key ministries. The Ministry of Health (MoH) is involved with the formulation of health policy, guidelines, strategies, and resource mobilization The. The President’s Office of Regional Authority and Local Government (PORALG) oversees the delivery of Primary Health Care (PHC). Prior to 2017/18 funds for health services delivery at primary health care were channeled through the districts headquarters who would allocate and spend the funds on behalf of health facilities. In 2017/18 a new policy of Direct Health Financing was instituted, all funds for service delivery for PHC are allocated directly to facilities from the Ministry of Finance and Planning (MoFP).” |  |
|  | **Method** | | |
| 1 | What are the confounding factors and how do these factors affect the results? For example, FFARS was also introduced in parallel to DHFF. How does this affect the interpretation of the results? Are the effects statistically significant? | Thanks, DHFF and FFARS was introduced in parallel, while DHFF is a policy of directly financing the primary health facilities. FFARS is an accounting and reporting system used to manage the funds disbursed directly to health facility bank accounts. A statement has been added, **“…..FFARS is an information financial management system used for accounting and reporting of funds disbursed directly to health facility bank accounts”** | **Pg.3** |
| 2 | The statement in Data management and analysis section is inconsistent. I pasted the sentence below: Data from four years was analyzed: one year before the implementation of DHFF (2015/16) and three years after (2016/17, 2017/18, 2018/19, and 2019/20). One suggestion is to consider regression analysis by considering the data at sub-region level: e.g.: 26 regions, 139 Districts, 185 Councils, 570 Divisions, 3956 Wards or 12319 villages. | Thanks the section has been edited to remove the unclear part. Inferential analysis was also done…. “Further inferential analysis was done using correlation and regression analysis to quantify the relationship between fund availability and health commodity availability”.  The data are presented in Figure 2 and table 6 | **Pg. 6**  **Pg. 11 and 12** |
| 3 | Further, the authors should consider collecting and showing data before 2015/16 to examine the trend in the outcome variables. | Thanks, unfortunately the data are not available, there was no financial management information system at PHC prior to DHFF. We have stated this as a limitation… “while interpreting the findings of this study it is important that such factors are taken into consideration that some of key source of health commodities funding might not have been captured in its entirety in this study. There was no financial data by facility levels prior to DHFF in 2015/16 to 2016/17, this limits the discussion of the effect of the health financing prior to DHFF policy”… | **Pg.13** |
| 4 | The authors should explain more about health commodities. This is not intuitive to me. What do health commodities include? | Thanks, a statement on explaining health commodities has been added… “Health commodities are items required for the delivery quality health services, including medicines, vaccines, medical supplies such as contraceptives dressings, needles and syringes, and laboratory/diagnostic consumables..” | Pg 3 |
| 5 | Measure for "health commodities availability" was not well defined. | Thanks further elaboration has been provided regarding the measure of health commodities availability… “The eLMIS database, provided data on the supply levels of 312 health commodities from MoH essential commodities tracer list. The MoH essential commodities tracer list was used to measure annual availability of health commodities at health facilities.” | **Pg.6** |
|  | **Results** | | |
| 1 | Table 1: there were huge reductions in health commodities budget from 2018/2019 to 2019/2020. In my opinion, the results “With the overall spending for 2017/18 being 20% of the total budgets while 27%, 80% and 96% for 2020/21” was mainly driven by a reduction in budget. I am not sure how to interpret the results. Does this mean inefficient planning at the beginning? Similar comments apply to **Table 2.** | Thanks, there has been bottlenecks in the interoperability of PlanRep and FFARS that limits analysis if budget and disbursement data…A statement has been added on the limitation section…….. “This paper has only analyzed expenditure data on health commodities, the inconsistencies in transferring budget data from PlanRep to FFARS due to the ongoing interoperability bottlenecks, did not allow the incorporating budget and disbursement data”….. | **Pg. 13** |
| 2 | Furthermore, were all the budget used efficiently? The authors mentioned about adherence to expenditure guidelines. But, more information and discussion should be provided. | Thanks, as explained above we have used ONLY expenditure  . |  |
| 3 | Table 3: this table is difficult to read. Please consider to change the format or use a figure to show the trend. | Thanks, table 3 now table 3 has been improved to improve reading and interpretation | **Pg.9** |
| 4. | Figure 1: "health commodity availability" was not well defined. The authors claimed “After DHFF implementation, this increased each year to 69% availability in 2016/17 and 76% availability in 2017/18.” It seems to me that the authors wanted to claim a casual impact here. But in my opinion, this statement is not meaningful. There was already an increase before the introduction of DHFF, from 64% to 69%. And furthermore, there could be other things happening at 2016/2017. This goes back to the issue of the statistical method. | Thanks, health commodities availability has been defined, further inferential analysis has been done to find out if there was a link between financial availability and the health commodity availability. Clearly stated the limitation of this study to allow readers to interpret the result with care and consider further studies in future. |  |
| 5 | Because the statistical method is not robust, the results cannot support many of the claims in the Discussion. | Thanks, the analysis has been improved, discussion language tone lowered to indicate that this is a baseline information in which to build further studies… | **Pg. 12-13** |
| 6 | Overall, the language is fine. But, additional language editing and proof reading are required as there are some typos and grammatical errors | Thanks, extensive editing has been done. |  |

**Journal Comments**

| **SN.** | **Reviewers Comments** | **Response** | **Page** |
| --- | --- | --- | --- |
|  | **Journal Requirement** | | |
| 1 | 1. Please send a completed 'Competing Interests' statement, including any COIs declared by your co-authors. If you have no competing interests to declare, please state "The authors have declared that no competing interests exist". | Thanks, a statement…. “all authors declare that they have no competing interests” ….. Has been added | **Pg. 14** |
| 2 | Please amend your detailed Financial Disclosure statement. This is published with the article. It must therefore be completed in full sentences and contain the exact wording you wish to be published.   a) Please clarify all sources of funding (financial or material support) for your study. List the grants (with grant number) or organizations (with url) that supported your study, including funding received from your institution. b) State the initials, alongside each funding source, of each author to receive each grant. c) State what role the funders took in the study. If the funders had no role in your study, please state: “The funders had no role in study design, data collection and analysis, decision to publish, or preparation of the manuscript.” | Thanks, the Financial Disclosure statement now leads…  “USAID- Global Health Supply Chain – Technical Assistance Tanzania for financial support of this work. But The funders had no role in study design, data collection and analysis, decision to publish, or preparation of the manuscript” ….. | **Pg.14** |
| 3 | In the online submission form, you indicated that "Data will be available from authors upon request". All PLOS journals now require all data underlying the findings described in their manuscript to be freely available to other researchers, either 1. In a public repository, 2. Within the manuscript itself, or 3. Uploaded as supplementary information. This policy applies to all data except where public deposition would breach compliance with the protocol approved by your research ethics board. If your data cannot be made publicly available for ethical or legal reasons (e.g., public availability would compromise patient privacy), please explain your reasons by return email and your exemption request will be escalated to the editor for approval. Your exemption request will be handled independently and will not hold up the peer review process, but will need to be resolved should your manuscript be accepted for publication. One of the Editorial team will then be in touch if there are any issues. | The data will be made publicly available |  |
