# Supplementary material for: Understanding the implication of direct health facility financing on health commodities availability in Tanzania
Source: PLOS Glob Public Health. 2023 May 8;3(5):e0001867. doi: 10.1371/journal.pgph.0001867 (PMC10166559; doi:10.1371/journal.pgph.0001867)
Supplement: Attachment — Submitted filename: Reviewer comments 1& 2.docx [file pgph.0001867.s002.docx]

| **Reviewer 1** | | | |
| --- | --- | --- | --- |
| **SN.** | **Reviewers Comments** | **Response** | **Page** |
| 1. | Thank you for addressing my comments in the revised version. I have minor concerns on the statistical analysis in Figure 2 and Table 6.  **Figure 2:** Using regression analysis in the revised is not necessary here. There are only 3 observations in each graph so there is very limited statistical power, the R-squared and coefficients does not mean a lot. In addition, these observations are time-series data, so the independence assumption of linear regression is not satisfied: health care facilities can budget their expenditure and medicine purchase so the data points are not independent observations. Further more, only the correlation was discussed in the manuscript, and the regression is not explained in the text.  My recommendation is to choose from one of the two following options:  1. report the aggregate trend by facility type, do not use regression analysis  2. If you prefer regression analysis, the observations should be individual health facilities. Then you have 5,143 facilities times 3 years = 15,429 observations. The independent variables can be year dummies, facility type, urban/rural, etc. | Thanks, we have incorporated the suggestion figure 2 now is reported ass aggregate trend data by facility type.  Figure | **Pg.11** |
|  | Table 6: Please report number of Rural and Urban health facilities in the table. | Thanks, we have reported table with urban rural facilities | **Pg. 11** |

**Reviewer 2**

| **SN.** | **Reviewers Comments** | **Response** | **Page** |
| --- | --- | --- | --- |
|  | **Minor Comments** | | |
| 1 | Please do another round of proofreading. I can still identify many typos and grammar errors, which affect the readability of the manuscript. | Thanks, Proof reading has been done throughout the manuscript | **Several pages** |
|  | “The complimentary funds (user fees and insurance) contributed about 20%) less than the 50% required by the cost sharing guideline.”  Extra “)”. |  |  |
|  | “In 2017/18 a new policy of Direct Health Financing was instituted,”  When a time phrase adds information to an independent clause or sentence that follows it, it should be followed by a comma.  The next sentence is an independent sentence. Full stop should be used after this sentence. |  |  |
|  | “In addition to implementing DHFF and FFARS, the Government, has also developed web based planning and budgeting tools PlanRep to assist in strategic planning and resource allocation.”  Extra comma after “the Government”. |  |  |
|  | “Strong health systems are advocated as a tool for accelerating UHC.(13)”  Citation should be before the full stop. |  |  |
|  | “The availability of health commodities in 2018/19 was above the national target of 80% availability of tracer medicine at hospital level, at health center and dispensary the availability was closer to the target However, at all levels, the availability decreased in the second and third year of analysis.”  Misuse of and missing full stop. |  |  |
|  | Stata, not stata. Capital “S”. |  |  |
|  | “The President’s Office, Regional Administration and Local Government (PORALG) is responsible for service delivery in primary health care facilities through…” PORALG has been explained in the Background. No need to repeat. |  |  |
|  | Explain “eLIMIS” at its first appearance in section "Sampling procedures and sample size" |  |  |
|  | **Major Comments** | | |
|  | **Results** | | |
| 1 | Under "Data management and analysis", please provide more information about correlation analysis and regression analysis. What tests were used for correlation analysis? For regression analysis, what were the control variables/confounders? Were linear regression used or other type of regression used? | In the current analysis we used two sample test of proportion to identify if there was any significant difference in the health commodity availability between rural and urban... This statement has been added | **Pg. 5** |
| 2 | Can I consider introducing FFARS as part of the DHFF reform? Or FFARS is something independent, but happened to be implemented at the same time with DHFF? | Thanks, the introduction of FAARS is part of the DHFF reforms… A sentence has been added….. *“To enable the operationalization of the DHFF the GoT introduced Facility Financial and Accounting System (FFARS) an electronic solution for improving financial management at facilities level”* | **Pg. 3** |
| 3 | Table 2: any explanation why “other health service expenditure” increased for Council Hospital but decreased for Health Center? | Thanks as stated in the discussion, some of the reasons might be;   1. Failure to adhere to cost sharing guideline that directs that, 50% of the complimentary funding should be allocated for Health commodities 2. The covid 19 pandemic might have forced the health system to spend funding elsewhere, health education and preventive services such as WASH interventions |  |
| 4 | Table 3: looking at the absolute term, expenditure increased more for urban area compared to rural area. Why? Was rural area prioritized at the beginning? | **Thanks,** Though not analyzed by this study, as stated above, this could be related to covid-19 expenditures, since urban areas was targeted for preventive and health promotion interventions |  |
| 5 | “facilities from USD$ 12.1 Mil (22.2% of the total health expenditure) in…” Typo? Should be 20.2%. | Thanks, that has been rectified.. | **Pg. 8** |
| 6 | For the following results: “Further examination of the correlation between health commodities availability and availability of funds, revealed that, there was a very strong positive correlation between percentage of health commodities expenditure and availabilities of medicines at the hospital level (R=1) and dispensary (R= 0.68). However, a negative correlation was observed between percentage of health expenditure on health commodities and availability of health commodities (R= - 0.94) (Figure 2).” Is this part done by regression analysis? If this is from regression, please show the regression results in a table, including number of observations, coefficient, p-value, and 95% confidence interval. The authors can consider to put the Table in the Appendix/Supplementary Material. | Thanks, since health care facilities can budget their expenditure and medicine purchase so the data points are not independent observations. So the independence assumption of linear regression is not satisfied. We therefore believed the best option was to report the aggregate trend by facility type. | **Pg.11** |
| 7 | Figure 2 is confusing. Based on Figure 2, it seems that the authors ran the regression just using 3 observations at the yearly level. Please clarify. The regression analysis should use the data at disaggregated level. It is uncommon that a regression analysis shows R-square = 1. | Thanks, the figure has been omitted, the data are reported as aggregate trend of health commodities expenditure and availability by health facility levels | **Pg.11** |
| 8 | Are the results from Table 6 derived from t-test?  It seems counter intuitive that medicine availability decreased over time. I would expect DHFF increase the availability. Any reason? Besides, how was health commodity availability calculated? For example, for medicine, is it that you take the ratio between the available medicine and the total number of medicine (312)? | Thanks, the new figure six is reported from two sample test of proportion  Thanks, the health commodities availability is a proportion of available tracer medicine over the expected available health commodities i.e. the total number of the tracer medicine (32) | **Pg.6** |
|  | **Discussion** | | |
| 1 | The authors claimed that “This study findings indicate that there is potentiality in DHFF improving the availability of health commodities”. Which part of the results can support this? Is it because of the increased health expenditure over time? But health commodity availability decreased in the recent three years. Please clarify. | Thanks, the sentence has been edited to read as per the support of the results… This study findings indicate that there is potentiality in DHFF improving the visibility of health commodities funding at the health facilities” The DHFF has enabled the analysis of health commodity funding over time | **Pg.11** |
| 2 | “The findings of this study have indicated that during the same period of implementing DHFF, there has been increased availability of health commodities measured by tracer medicine.” This statement is inconsistent with results from Table 5. | Thanks, we have rectified the sentence to indicate that DHFF has increased visibility and not availability. | **Pg. 11** |
