# Supplementary material for: Understanding the implication of direct health facility financing on health commodities availability in Tanzania
Source: PLOS Glob Public Health. 2023 May 8;3(5):e0001867. doi: 10.1371/journal.pgph.0001867 (PMC10166559; doi:10.1371/journal.pgph.0001867)
Supplement: Attachment — Submitted filename: Reviewer comments.docx [file pgph.0001867.s003.docx]

| **Reviewer number 1** | | | |
| --- | --- | --- | --- |
| **SN.** | **Reviewers Comments** | **Response** | **Page** |
| 1 | In the Abstract – Results and the Main manuscript – Discussion, the authors mentioned “visibility” in the first sentence. This is confusing and not inline with the objective of the study. The objective of the paper is to understand the impact on health commodities availability. The author may mention “visibility” in the manuscript, but “visibility” should not be the first thing mentioned as this is not part of the study objective.  The authors are not obligated to say that the intervention increased commodity availability. The authors just need to reported the results. | Thanks, the statement has been edited now reads…”This study findings indicate that there has been an increase in allocation of funds for health commodities. The allocation of funds for health commodities increased over the past three years….. The visibility has been added to the end….. now reads….**…** “There is potentiality in DHFF improving visibility and tracking of funding for health commodities...”. | **Pg.2** |
| 2 | Figure 1: the authors should remove data 2016/17. In the “Data management and analysis”, the authors mentioned data to be used are from 2017 to 2021. It is inconsistent to include data 2016/2017 in the figure. Alternatively, the authors should include a footnotes under Figure 1 to explain why there is data from 2016/2017 here. | Thanks, I have added the following statement to clarify the use of 2016/17 data for health commodities….. “The analysis for health commodities included 2016/17, 2017/18, 2018/19, 2019/20 and 2020/21. The year 2016/17 was used as baseline before the launch of DHFF. However, the expenditure data were only available from 2017/18 when DHFF was introduced” | **Pg. 5** |
| 3 | The authors explained about the difference between rural areas and urban areas (Table 3 and Table 6) in their response letter but not in the manuscript. The authors should put this in the Discussion. This is important as potentially there is equity implication. | Thanks, a statement has been added in the discussion section… “Looking at the absolute term, health commodity expenditure increased more for urban area compared to rural area. Though not analyzed by this study, this could partly be explained by the covid 19 pandemic. The pandemic might have forced the health system to spend funding elsewhere, such as health education and preventive services e.g., WASH interventions. This intervention targeted urban areas due the high prevalence rate of covid19 in urban areas compared to rural areas. Future studies could focus in this area to explore the equity in financing between rural and urban areas… | **Page 12** |
| 4 | Table 5: the absolute Health commodities expenditure increased or increased first then decreased. However, the Average availability of medicine kept decreasing for all type of Facilities. The authors should discuss about this in the manuscript about the potential reasons and the implication on health. | “Similar trends are observed, when examined by level of health services. The funding is steadily increasing at hospital levels but fluctuating at health centers and dispensaries. It remains an empirical question that needs further exploratory studies to identify the underlying causes. One underlying cause could be inadequate or low skilled accountants in lower-level facilities leading to poor data quality. A previous study on the review of the implementation of FFARS in Tanzania indicated existence of inadequate accountants, one district had only four accountants, that oversees about 100 facilities. Another study in Tanzania on the use web-based planning and budgeting tools, indicated that shortage of ICT equipment and access to reliable internet complicates the utilization of such innovative systems, more so in rural areas….” | **Pg 12** |
| 5 | There are still language and formatting issues. I will leave these to the journal editing team. | Thanks, extensive editing has been done.. |  |
